# Matthias Ehrhardt, Michael Günther, Wil Schilders: Erfolgsformeln – Anwendungen der Mathematik: Books4You, ’s Hertogenbosch 2021, 212 Seiten

**DOI:** 10.1007/s00591-022-00325-y

**Published:** 2022-07-29

**Authors:** Ludger Hartmer, Harald Krawczyk

**Affiliations:** 1 Adenauerstraße 1A, 59759 Arnsberg, Deutschland; 2 Institut für Methodik der Fernerkundung, Abteilung Photogrammetrie und Bildanalyse, Deutsches Zentrum für Luft- und Raumfahrt e. V. (DLR), Rutherfordstraße 2, 12489 Berlin-Adlershof, Deutschland

*„Mathematik ist wie Sauerstoff. Wenn sie da ist, bemerkt man es nicht. Wenn sie nicht da wäre, könnte man nicht ohne sie leben.“*

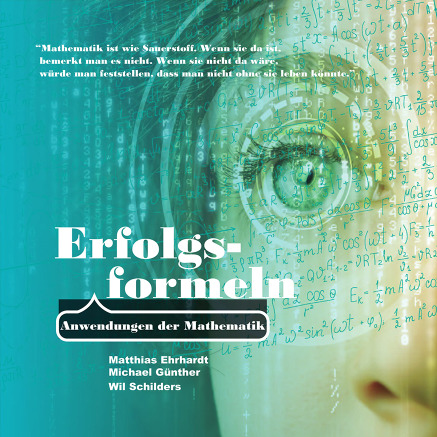


Dieses von Lex Schrijver stammende Motto steht dem im September 2021 erschienenen Buch *Erfolgsformeln – Anwendungen der Mathematik* von Matthias Ehrhardt, Michael Günther und Wil Schilders voran, das sowohl als Download^1^ als auch als kostenloses gedrucktes Exemplar erhältlich ist. Das Buch richtet sich den Herausgebern zufolge „nicht nur an Mathematikinteressierte, sondern vor allem an erklärte „Nicht-Zahlenmenschen“ und Formeljongleure im Alltag“. Dementsprechend wird es auch hier in den „*Mathematischen Semesterberichten*“ nicht von Mathematikern rezensiert.

Für den erstgenannten Referenten (L. H.) als Mediziner waren vor allem die ersten Kapitel mit Themen der angewandten Mathematik aus dem medizinischen Bereich von Interesse.

Die beiden Herausgeber Prof. Matthias Ehrhardt und Prof. Michael Günther wollten Interessierten die Aufgaben und Arbeitsweisen in der angewandten Mathematik nahebringen und konnten dazu auf die Erfahrungen ihres Kollegen Prof. Wil Schilders zugreifen, der als Gastprofessor von der Technischen Universität in Eindhoven, Niederlande bei ihnen an der Bergischen Universität Wuppertal weilte und 2014 ein ähnliches Buch veröffentlicht hatte. Also taten sie sich zusammen, konnten neun etablierte Wissenschaftlerinnen und Wissenschaftler der angewandten Mathematik interviewen und viele andere dazu bewegen, Aufsätze zu mathematischen Lösungswegen in ihrem jeweiligen Arbeitsfeld zu schreiben. Die Themenbereiche sind breit gestreut von der Modellierung in der Epidemiologie, soziologischen Themen bis zur künstkichen Intelligenz und anderen Themengebieten.

Wie das so ist, wenn viele Autoren und Autorinnen mitgearbeitet haben, sind die Kapitel sehr unterschiedlich in ihrer Ausführlichkeit und Verständlichkeit. Auch die Anforderungen an die mathematische Vorbildung sind unterschiedlich hoch. Zum Trost gibt es einen Satz im Interview von Dr. Thomas Hahn (seit 2011 Chief Expert Software bei der Siemens AG): „Und mein letzter Hinweis ist, dass man in der Mathematik auch nicht immer alles verstehen muss.“ Das passt zu diesem Buch: Es ist kein Lehrbuch; es will Interessenten dazu bringen, sich näher mit der Materie zu befassen und das in ihre Berufsplanung einfließen zu lassen. Solche Interessenten können Abiturienten/Abiturientinnen sein oder Naturwissenschaftler als Seiteneinsteiger, Studienanfänger oder Lehrer, die ihre Schülerinnen/Schüler gut beraten wollen.

Natürlich ist dieses Buch kein Lehrbuch für Mediziner und will es auch nicht sein. Beim ersten Kapitel wird nicht klar, wer diesen Aufsatz geschrieben hat. Mal wird Frau Dr. Maria Vittoria Barbarossa zitiert, in welcher persönlichen Situation sie war, als die COVID-Pandemie aufkam und wie sie und andere zusammen erste Modellierungen der Infektionsverbreitung entwickelt haben. Dann scheint sie selber das SIR-Modell einer Pandemie zu erklären: wie sich die Veränderungen der Zahl der Empfänglichen $S^{\prime}(t)$, Infizierten $I^{\prime}(t)$, der Genesenen/Verstorbenen $R^{\prime}(t)$ und die Basisreproduktionszahl $R_{0}$ mathematisch beschreiben lassen und wie sich diese Werte bei verschiedenen Infektionskrankheiten auswirken. Dann folgen Zitate, wie sie selber zur angewandten Mathematik gefunden hat.

Im zweiten Kapitel geht es um die Zuverlässigkeit von Corona-Schnelltests. Die wird zwar vorgerechnet, anschaulicher wäre aber gewesen, zusätzlich eine Wahrheitsmatrix (Konfusionsmatrix) und den Fachbegriff „positiver Vorhersagewert“ (positive predictive value $PPV$) zu verwenden. Ich vermisse einen Verweis auf die drastisch bessere Spezifität der PCR auch bei niedriger Inzidenz und eine mathematische Begründung dafür (gemeinsame Bestimmung zweier verschiedener Nukleotidsequenzen).

Das dritte Kapitel über Inzidenzzahlen ist zwar sehr interessant und ausführlich, aber eher geeignet für Leser, die schon etwas Mathematik studiert haben. Ausgleichsrechnungen gehörten bei mir 1969 jedenfalls nicht zum Abiturwissen.

Im vierten Kapitel geht es um die Effekte nicht-pharmazeutischer Interventionen bei Pandemien. Es wird gezeigt, wie es damit unter Zuhilfenahme von Modellierungen möglich ist, einen Pandemieverlauf so zu steuern, dass die Zahl der gleichzeitig Infizierten das Gemeinwesen nicht überfordert.

Im fünften Kapitel geht es um die Quantifizierung der Wirksamkeit von Impfstoffen. Zuerst werden die immunologischen Grundlagen erklärt. Es wird der Begriff der „verlorenen Lebensjahre“ genannt. Die folgende Rechnung mit Regressionsmodellen übersteigt wieder mein Abiturwissen von 1969. Dann steht da ein wichtiger Satz: „Die Wirksamkeit von Impfstoffen in klinischen Studien (engl. vaccine efficacy, $VE$) ist nicht zu verwechseln mit der Impfstoffeffektivität (engl. vaccine effectiveness, $VE_{eff}$), der Wirksamkeit des Impfstoffes unter klinischen Alltagsbedingungen.“ Das wird ausführlich mathematisch ausgeführt. Es wird am Beispiel u. a. der Masern auch der Begriff der „Herdenimmunität“ mathematisch untersucht. Leider ist der Begriff für den Verlauf der Sars-CoV2-Pandemie ungeeignet, weil diese Viren zu stark mutieren.

Im sechsten Kapitel geht es die Optimierung von Dosierung und Therapieintervall einer zytostatischen Behandlung. Diese Therapien brauchen zur Antitumorwirkung eine gewisse Mindestdosierung, die aber die Blutneubildung im Knochenmark schädigt. Der Pfad zwischen zu schwacher therapeutischer Wirkung und zu starker toxischer Nebenwirkung ist nur schmal. Es werden Ergebnisse demonstriert, wie man diesen Pfad mathematisch beschreiben und planen kann. Am Schluss des Kapitels steht ein wichtiger Satz: „Wer an der Mathematik mit Anwendung Pharmazie Interesse hat, sollte ein grundständiges Mathematik-Studium wählen, z. B. mit Nebenfach in Richtung Biologie, oder falls möglich Pharmazie. Wichtig ist eine solide Grundausbildung; spezialisieren kann man sich später.“

Das Buch eignet sich gut zur Berufsplanung von Mathematikstudenten oder wissenschaftlichen Seiteneinsteigern, die sich für angewandte Mathematik interessieren. Es eignet sich nicht zur Fortbildung von Medizinern, ist dafür aber auch nicht geschrieben worden. (L. H.)

Der folgende zweite Teil der Besprechung wurde vom zweitgenannten Rezensenten (H. K.) verfasst.

Dieses Buch wendet sich an eine interessierte Leserschaft, um dieser einen Einblick in die vielfältigsten Anwendungsfelder der Mathematik in unserem Alltag zu ermöglichen. Es ist kein Lehrbuch, es erfordert keine Spezialkenntnisse der Mathematik und ist somit sowohl für mathematische Laien als auch Fortgeschrittene geeignet. Auch Spezialisten können dieses Werk durchaus mit Interesse lesen, auch wenn sie keinen unmittelbaren Wissenszuwachs erwarten sollten.

Ziel des Buches ist es, an Hand einer Vielzahl von Beispielen, nicht unmittelbar aus dem täglichen Leben, aber immer unseren Alltag betreffend, die Rolle und Nützlichkeit von mathematischem Wissen aufzuzeigen.

Da dieses Buch von Mathematik handelt, hier zunächst einige mathematische Zahlen und Fakten. Das Buch hat ca. 195 Textseiten, 18 Kapitel und 64 Beiträge sowie 9 Interviews. Die durchschnittliche Länge eines Artikels beträgt also ungefähr 3 Seiten. Daran sieht man schon, dass die Thematik sehr breit gefächert ist. Sie reicht von Medizin, Chemie über Kunst und Musik bis hin zu einem Kapitel über Kriminologie. Angenommen, man veranschlagt für ein bewusstes Lesen ca. 5 Minuten pro Seite, dann benötigt man für ein komplettes Durchlesen ungefähr 16–17 Stunden. Durch die Vielfalt der behandelten Themen kann man dieses Buch aber problemlos auszugsweise und mit Unterbrechungen lesen, so dass bei einer Beschäftigungsdauer von 1–2 Stunden pro Tag ein Urlaub mit diesem Buch sehr gut verbracht werden kann.

Im Folgenden sollen einige der Kapitel und Artikel kurz gestreift werden, um beim potentiellen Leser das Interesse zu wecken, womöglich doch tiefer in die Welt der Mathematik einzutauchen.

Wahrscheinlich geschuldet der aktuellen Covid-19 Situation beginnt das Buch mit einem Kapitel über Epidemiologie. Der erste Artikel beschäftigt sich auf 3 Seiten mit der Erklärung von Infektionsmodellen, also der Frage, wie schnell sich ein Virus in der Bevölkerung ausbreitet. Beschrieben wird das an Hand des inzwischen weit genutzten sogenannten „SIR-Modells“, eines Klassikers schon aus den 60-er Jahren des letzten Jahrhunderts. $SIR$ steht dabei für $S$ wie Susceptible (empfänglich für das Virus), $I$ wie Infected (infiziert mit dem Virus) und $R$ wie Recovered (genesen oder gestorben). Die hierfür benutzten gewöhnlichen Differentialgleichungen werden verständlich erklärt, und ein Rechenbeispiel graphisch anschaulich dargestellt. Die auch in den Medien oft erwähnte Basisreproduktionszahl wird erklärt. Ein extra Absatz widmet sich dann Erweiterungen dieses Grundmodells, um Aussagen über die Virusausbreitung zu verfeinern. Obwohl der Artikel nicht über den Inhalt des Wikipedia-Eintrags hinausgeht, lohnt es sich durchaus beide vergleichend zu lesen, da dadurch das Verständnis der Methodik vertieft werden kann.

Der darauf folgende Artikel „Die Verlässlichkeit von Covid-19 Schnelltests“ gibt einen interessanten und konzentrierten Kurzeinblick in das Gebiet der bedingten Wahrscheinlichkeiten mit der Erläuterung des Bayes-Theorems an Hand der Erläuterung des Zusammenhangs zwischen falschen Testergebnissen und einer tatsächlichen Erkrankung. Es wird vorgerechnet, wie uns die Intuition der Vorstellung von Wahrscheinlichkeiten einen gewaltigen Streich spielen kann. Das ist spätestens seit der Diskussion über HIV-Tests bekannt, ist aber durchaus Wert hier noch einmal an einem aktuellen, graphisch sehr instruktiv illustrierten Beispiel aufgefrischt zu werden.

Der nächste Artikel widmet sich dem Thema „Robuste Inzidenzzahlen“, einem Schlagwort, das lange Zeit als 7‑Tage-Inzidenz in aller Munde war. Dieser Beitrag bedarf allerdings von Seiten des Lesers schon etwas erweiterter Kenntnisse der Mathematik sowie deren Notation. Umgang mit Logarithmus und Exponentialfunktion sowie Matrizenrechnung sind zum Verständnis erforderlich. Der Hintergrund bzw. die Motivation der durchgeführten Berechnungen erschließen sich aber ganz gut aus dem erklärenden Text. Es wird nämlich ausgeführt, dass die 7‑Tage-Inzidenz kein sonderlich robuster Parameter ist, da er stark durch äußere Gegebenheiten wie die Zahl der Tests und die Qualität der Kontaktnachverfolgung beeinflusst wird. Der Autor erläutert dann mathematisch etwas anspruchsvoller ein alternatives Verfahren zur Schätzung der Infektionsbelastung an Hand des EPG-Indexes. Allerdings ist auf Grund der stark komprimierten Darstellung sowie einer Vielzahl von verwendeten Abkürzungen bzw. Formelzeichen ein schnelles und komplettes Durchdringen und Nachvollziehen der Methodik nicht ohne weiteres möglich. Aber wenn das Interesse bei dem einen oder anderen fortgeschritten Leser geweckt wurde, kann er durch ein mitgeliefertes Quellenverzeichnis sein Verständnis erweitern.

Schauen wir uns zur Abwechslung zwei Beiträge aus dem Kapitel Sport an. Beginnen wir mit dem Artikel „Der optimale Freiwurf im Basketball“. Hier wird ein – eigentlich physikalisches – Problem erläutert, wie beim Basketball ein Freiwurf zu auszuführen ist, der einen möglichst großen Spielraum an Abwurfparametern zulässt. Der Sportler visiert den Korb unter bestimmtem Winkel und Abwurfgeschwindigkeit an, um den Korb zu treffen. Dafür könnte er einen flachen oder auch steileren Wurf planen. Da seine Schätzung aber verständlicherweise nicht perfekt ist, geht es darum unter welchem Winkel eine möglichst große Abweichung vom optimalen Wurf noch zum Korb führt. Es wird somit eine Strategie entwickelt, die unter realen Bedingungen die Erfolgsquote deutlich erhöhen kann. Leider wird die Lösung nicht weiter ausgeführt, sondern nur darauf hingewiesen, dass die beste Strategie darin besteht, sowohl den Abwurfwinkel als auch die Abwurfgeschwindigkeit zu optimieren. Da es sich aber im betrachteten vereinfachten Modell um die Analyse reiner Wurfparabeln handelt, kann man dies gerne als Anregung nehmen, diese Aufgabe in einer Arbeitsgemeinschaft unter fachkundiger Anleitung ausführlicher zu untersuchen.

Im folgenden Beitrag „Das Geheimnis hinter einem erfolgreichen Endspurt im Radsport“ erläutert ein bei einem sehr bekannten Radrennstall (Shimano) angestellter Bewegungswissenschaftler, wie er alle ihm übermittelten Messdaten der Rennfahrer, sogenannte SRM-Daten, für eine optimale Zielsprinttaktik auswertet. Leider geht es auch hier nicht ins Detail, aber einige radsportaffine Leser werden das präsentierte Diagramm sicher sehr interessant und vielleicht sogar hinterfragenswert finden.

Da in diesem kurzen Abriss unmöglich alle Beiträge erwähnt werden können, abschließend noch ein Beispiel aus einem Gebiet, in dem man weniger Mathematik vermutet: aus der Musik zum Thema „Wie fair ist die Punktevergabe im Eurovision-Song-Contest“. Hier wird von den Autoren diskutiert, ob man aus dem Abstimmverhalten der einzelnen Länder erkennen kann, ob sich bestimmte Ländergruppen gegenseitig Punkte „zuschieben“, oder ob doch eine halbwegs „objektive“ Bewertung der Songs vorliegt. Dies erfolgt an Hand von Begriffen wie Nachbarschaft, Sprachvorliebe und ähnlichen. Dabei erklären sie, wie bei dieser Untersuchung Elemente der Graphentheorie zur Anwendung kommen können. Das sind Untersuchungen, wie man sie schon bei der Analyse von sozialen Netzwerken wie Facebook durchgeführt hat. Der Autor kommt zu dem Schluss, dass bei skandinavischen Ländern wahrscheinlich der ähnliche Musikgeschmack die Punktevergabe erklärt und Lena Meyer-Landrut ihren Wettbewerb zurecht und fair gewonnen hat.

Abschließend kann man sagen, dass dieses Buch eine Vielzahl von Themen in verständlicher und kurzer Weise darbringt. Das erforderliche Niveau zum Verständnis ist dabei durchaus unterschiedlich und reicht von rein verbaler Darstellung bis hin zu detailierteren mathematischen Erläuterungen. Insgesamt vermittelt das Buch einen interessanten Einblick in die Vielfalt der mathematischen Anwendungen für die unterschiedlichsten Gebiete der Gesellschaft. (H. K.)

## Footnotes

1 https://erfolgsformeln.uni-wuppertal.de/fileadmin/mathe/erfolgsformeln/Erfolgsformeln.pdf

